# TRP channels and breast cancer: the role of TRPs in the pathophysiological development

**DOI:** 10.3389/fmolb.2025.1528663

**Published:** 2025-02-26

**Authors:** Yu Zhang, Yanfeng Yi, Yinghao Shu, Xiaochen Ru, Shuaibing He

**Affiliations:** ^1^ Key Laboratory of Vector Biology and Pathogen Control of Zhejiang Province, School of Medicine, Huzhou University, Huzhou, China; ^2^ Department of Life Sciences and Health, School of Science and Engineering, Huzhou College, Huzhou, China

**Keywords:** TRP channel, breast cancer, calcium signaling, carcinogenesis, treatment

## Abstract

TRP channels play important roles in regulating various physiological and pathological processes, including the progression of cancer. Several TRP channels mediate tumour development. This review focuses on the role of TRP channels in the development of breast cancer, including their involvement in proliferation, apoptosis, autophagy, metastasis, and angiogenesis. TRP channels are associated with breast carcinogenesis and their role as potential therapeutic targets and prognostic biomarkers is under investigation. This review summarizes the reported effects of inhibiting or agonizing various TRP channel in breast cancer cells. Although there are relatively mature protocols for the treatment of breast cancer, its treatment is not currently a breakthrough, and therapies targeting TRP channels may be a developable strategy for it.

## 1 Introduction

Breast cancer ranks first in the global incidence of female malignant tumours and is highly heterogeneous, metastatic and drug-resistant (Nolan et al., 2023). The treatment approach currently employed is based on the principle of integrated treatment. This approach combines multiple therapeutic methods based on the biological behavior of the tumor and the physical condition of the patient. It takes into account both local and systemic treatments and mainly involves surgical resection, radiotherapy, chemotherapy, endocrine therapy, targeted therapy, and traditional Chinese medicine treatment (Chen X. et al., 2022). Nevertheless, the aforementioned conventional cancer treatment programs are associated with high recurrence rates and can have serious side effects. The side effects associated with endocrine therapies include irritability, vaginal dryness, irregular vaginal bleeding, and other perimenopausal-like syndrome manifestations. Nausea, gastrointestinal reactions, and dermal toxicity may ensue from targeted therapies. Myelosuppression and cardiotoxicity are common adverse effects of anthracyclines. Such symptoms frequently render it intolerable for patients to cease treatment, thereby jeopardizing the overall outcome (Abid et al., 2024). It is thus imperative to develop innovative molecular therapies that target a multitude of cancer-related cellular processes, thereby offering cancer patients a plethora of treatment options and efficacious alternatives in instances of suboptimal efficacy or intolerance.

In recent years, the rapid development of therapeutic directions such as biologic therapy and ion channel-targeted therapy has marked a new stage in the development of cancer-related research, and they may become potential targets for cancer treatment (Santoni et al., 2019). Ion channels are important regulators of cellular activity. Through pore-forming structures in the plasma membrane, ion channels selectively conduct ions, and one of its main regulation mechanisms is protein-protein interactions. Numerous physiological and pathological processes, including those involved in the development of cancer, are regulated by ion channels. These mechanisms include tumor growth, apoptosis, migration, invasion, and chemoresistance (Saldías et al., 2021). A growing body of evidence indicates that aberrations in various ion channels, which result in altered or defective bioelectric signals, may be pivotal in the pathogenesis of cancer. The development of a variety of tumors is associated with mutations or loss of expression and aberrant expression of voltage-gated ion channel genes (Sakellakis et al., 2024). Voltage-gated sodium channels directly influence cell cycle regulation, tumor cell metastasis, and intracellular calcium levels in breast, cervical, ovarian, and prostate cancers (Fraser et al., 2005). Modifications to voltage-gated potassium channels have been linked to apoptosis, motility, and growth regulation in a range of neoplastic diseases, including breast, melanoma, glioblastoma, and prostate cancer (Cammann et al., 2023; Payne et al., 2022). The TRP family of ion channels has been recognized as a significant contributor to the progression of breast cancer, distinguishing itself from other families of ion channels. And among them, TRP channel-mediated calcium changes play a prominent role in cancer, and dysregulation of multiple downstream effectors dependent on Ca^2+^ homeostasis has been linked to breast cancer proliferation, survival and invasion (Fraser et al., 2005).

Transient receptor potential (TRP) channels are permeable to calcium and are expressed in a variety of cell and tissues types in mammals. They play a crucial role in regulating the balance of physiological processes involving calcium and magnesium ions. Dysfunctions in ion channels can lead to various pathologies, including cancer (Kärki et al., 2020). Drugs that affect ion channels have significant potential for application. They can open or close ion channels, act as ion channel receptor agonists, antagonists, or alter enzyme activity. Over the past 2 decades, a growing body of evidence has strongly supported an inextricable link between TRP channels and cancer. Altered TRP channel expression can serve as a diagnostic aid in identifying evidence of cancer-related processes, including tumour progression, apoptosis inhibition, proliferation, migration, invasion and angiogenesis, and may even be a potential therapeutic target for tumours (Table 1) (Morelli and Amantini, 2022; Foster et al., 2023; Chen et al., 2023). This article reviews the TRP channels involved in breast cancer development and other physiological responses, and the related research on currently available TRP channel drugs, with the aim of providing new ideas for breast cancer treatment.

**TABLE 1:** Regulatory mechanisms of TRP channels.

Regulatory mechanisms	TRPC	TRPV	TRPP	TRPA	TRPML	TRPM	Reference
Voltage activation		TRPV1				TRPM2, TRPM4-5 TRPM8	Owsianik et al. (2007)
Temperature activation	TRPC5	TRPV1-4		TRPA1			Li et al. (2018)
Mechanical activation	TRPC1 TRPC6	TRPV4				TRPM4 TRPM7	Landini et al. (2022), Marini et al. (2023)
Ligand activation	TRPC1	TRPV1				TRPM2 TRPM4 TRPM5	Koivisto et al. (2022)

## 2 TRP channels

TRP channels constitute a non-selective superfamily of cation channels, and mammalian TRPs can be divided into six subfamilies based on differences in amino acid sequence (homology): TRPC (canonical; TRPC1-7), TRPV (vanilloid; TRPV1-6), TRPP (polycystin; TRPP2, TRPP3, TRPP5), TRPA (an-kyrin, TRPA1), TRPML (mucolipin; TRPML1-3) and TRPM (melastatin; TRPM1-8). TRPN (NOMPC-like) is also included, but is only found in invertebrates and fish. The TRP channel is composed of four identical or similar subunits, each containing six transmembrane structural domains. A cationic pore is located between structural domains 5 and 6, which is mainly permeable to Ca^2+^, but also allows the passage of cations such as Mg^2+^, K^+^, and Na^+^. TRP channels are widely distributed in various tissues and cells. They regulate changes in cation concentrations, which alter the strength of signals from corresponding pathways in the cell, leading to changes in cellular function.

The TRP channels, serving as pivotal cellular sensors, facilitate the transmission of information both intracellularly and extracellularly. Furthermore, they are also regulated by messenger molecules and compounds from inside or outside the cell, as well as by variations in temperature and osmotic pressure. Different TRP channels are regulated by different mechanisms, and many TRP channels can be regulated by multiple stimuli, including activation and inactivation, which is, opening or closing of the channel. Receptor activation can be via G protein-coupled receptors or tyrosine kinase receptors, respectively, through activation of phospholipase C, which leads secondary activation of TRP channels (Owsianik et al., 2007). Additionally, endogenous substances, exogenous small molecules, inorganic ions, and other compounds have the potential to act as ligands, thereby activating TRP channels. Some exogenous small molecules, compounds, inorganic ions, and endogenous substances can also act as ligands to activate TRP channels. The most extensively studied exogenous activator is capsaicin, which has been shown to specifically activate TRPVl channels (Li et al., 2018). Change in temperature can also directly activate a variety of TRP channels, and different temperature-sensitive TRP channels have different activation thresholds. The TRP channel subtypes activated by thermal stimulation include TRPVl, TRPV2, TRPV3 and TRPV4, whereas the TRP channel subtypes activated by cold stimulation are TRPM8 and TRPAl (Landini et al., 2022). Other mechanisms include osmotic stress reduction to activate TRPV4 and TRPM3, pH < 5.9 to activate TRPV1, mechanical stretch to activate TRPCI and TRPPl, and a number of TRP channels show some voltage-dependent activation properties, such as TRPVl, TRPM4, TRPM5, and TRPM8 (Marini et al., 2023). In contrast to the activation of TRP channels, little is known about the mechanism of inactivation. Inactivation mechanisms include temperature inactivation, ligand inactivation, and others such as calmodulin, which can bind to ankyrin repeats, or intracellular ATP-mediated inactivation (Koivisto et al., 2022).

### 2.1 Ions mediated by the TRP channel

TRP channels are non-selective cation channels that transduce ions such as Ca^2^⁺, Na⁺, and Mg^2^⁺. Different TRP channels exhibit distinct permeabilit ratios (PCa/PNa) for Ca^2^⁺ and Na⁺. The TRPV5 and TRPV6 isoforms demonstrated a high degree of selectivity for Ca^2^⁺, exhibiting PCa/PNa values exceeding 100. Conversely, some isoforms exhibited PCa/PNa values below 10, while the TPRM4 and TRPM5 isoforms displayed minimal Ca^2^⁺ transduction. The activation of TRP channels is initiated by a number of different mechanisms, including changes in membrane potential, alterations in membrane phospholipids, phosphorylation, and the binding of specific ligands. This process results in an increase in cell membrane depolarisation, which in turn activates voltage-dependent ion channels, leading to a change in the concentration of intracellular calcium ions (Nilius and Owsianik, 2011).

The role of calcium channels in TRP channels is increasingly being studied and Ca^2+^ has been shown to play an important role. While the role of calcium in regulating most TRP channels is widely recognized, there are still numerous aspects regarding the regulation mechanisms and mutant pathogenesis that require further elucidation. However, there have been many studies that have progressively explored it in depth. Taking the study of TRPC3/6 channels as an example, Wenjun Guo et al. determined that TRPC3/6 channels have an inhibitory Ca^2+^-binding site CBS1 in the cytoplasmic region through structural analyses and functional validation (Guo et al., 2022). At elevated calcium concentrations, this site binds calcium and facilitates the close alignment of multiple cytoplasmic structural domains, thereby impeding the flux of cations from the intracytoplasmic cavity of the TRPC channel into the cytoplasm. When the concentration of calcium ions is low, the calcium ions dissociate from this site, resulting in a more relaxed structure of the cytoplasmic region. This allows for the flow of ions from the intracytoplasmic cavity to the cytoplasmic region. This inhibitory site provides negative feedback regulation of TRPC channels by Ca^2^⁺. Additionally, TRPC channels possess an activated Ca^2^⁺ binding site, designated as CBS3, within their transmembrane region. The superimposed effects of inhibitory and activating Ca^2^⁺ binding sites result in a complex response of TRPC channels to intracellular calcium ion concentrations. An imbalance in the regulation of these two binding sites can lead to the mediation of a variety of pathologies. To illustrate, the TRPC6 GOF mutation identified in patients with focal segmental glomerulosclerosis (FSGS) impairs the function of the inhibitory CBS1 in the cytoplasmic region of TRPC6, yet does not affect the function of the activating The sustained activation of the mutant channel in the presence of Ca^2+^influx through positive feedback, resulting from the activation of CBS3, leads to an overload of Ca^2+^ ions in renal pedunculated cells. This ultimately triggers the development ofFSGS. The mechanisms by which other TRP channels regulate Ca^2+^ are distinct. While calcium-dependent regulation of TRP channels is essential, the exact mechanisms remain to be elucidated.

### 2.2 Physiological and pathophysiological functions of TRP channels

TRP channels are widely found in a variety of mammalian tissues. They can sense temperature, mechanical pain, chemical stimulation, hearing, smell, taste, and participate in the regulation of muscle contraction, transmitter release, cell proliferation, cell differentiation, gene transcription, apoptosis, cell death and many other physiological functions (Qiao et al., 2024). TRP channels are found not only in nervous tissues such as brain, spinal cord and peripheral nervous system, but also in non-nervous tissues such as heart, kidney, testis, lung, liver, spleen, ovary, intestine, prostate, placenta, uterus and bladder (Sánchez-Hernández et al., 2024).

In recent years, TRP channels have been gradually investigated in pain-mediated, cardiovascular diseases, neurodegenerative diseases, and the relationship between TRP channels and various types of cancer. For example, many inflammatory mediators can interact with TRPVl channels and promote the sensitization of sensory nerves in pain signaling, mediating inflammatory pain and cancer pain (Chen W. et al., 2022). Two missense mutations of TRPV4 (R616Q and V6201) lead to gain-of-function mutation of TRPV4, which significantly increases the open probability of TRPV4 channel, resulting in the development of bone metabolic disorders like scoliosis (Das and Goswami, 2019). In addition, The inhibition of neointimal hyperplasia by a TRPC1 specific antibody (T1E3) further supports the involvement of TRPC1 in the pathogenesis of vascular obstructive diseases, such as atherosclerosis (Kumar et al., 2006). Hypoxia can increase the expression of TRPC1 in pulmonary artery smooth muscle cells, while downregulation of TRPC1 can reduce the proliferation of pulmonary artery smooth muscle cells (Wang et al., 2008).

Dysfunctional TRP channels may generally lead to disease through one or more of the following mechanisms: (1) As a second messenger, Ca^2+^ plays an important role in the body, and most TRP channels are permeable to Ca^2+^. The functional changes of TRP channels will lead to Ca^2+^ disorder and imbalance, which will further have a strong impact on a variety of cell and system functions. For example, gain-of-function mutations in TRPC6 lead to massive influx of Ca^2+^ and activation of T cytokines, which ultimately lead to cardiac hypertrophy (Kuwahara et al., 2006). (2) As a multi-gated channel, TRP channels can sense the changes of various physical and chemical factors inside and outside the cell and initiate the self-protection behavioral response of cells, tissues and organs. Errors in these sensory inputs may lead to multiple forms of cellular and somatic sensory dysregulation (Marini et al., 2023). (3) Some TRP channels have different permeability to different types of cations, such as Mg^2+^ and Ca^2+^. Dysfunctional TRP channels lead to disturbances in the balance between Mg^2+^ and Ca^2+^ in the body (Saldías et al., 2021). (4) TRP channels are found in the inner membrane of cells, and their dysfunction may lead to organelle dysfunction, such as lysosomal dysfunction caused by TRPML1 mutations (Thompson et al., 2007). (5) Some TRP channels are involved in the process of cell proliferation and growth and development. Therefore, channel dysfunction may lead to growth disorders and carcinogenesis (Bhaskaran and Smith, 2010). (6) TRP channels regulate the electrical activity of excitable cells such as the brain and heart. For example, TRPVl channels are expressed in the hippocampus and dentate gyrus, regions of the brain that readily activate periodic excitatory circuits that have been shown to be critical in seizure formation (Wang X. et al., 2022).

### 2.3 TRP channels and cancer

TRPs have been identified as a key factor in the development and progression of cancer. It affects the oxidative stress response of cells, cell survival, etc., through the permeation of Ca^2+^, Mg^2+^, etc. Changes in the function and expression of TRPs have a significant impact on cell proliferation as well as the susceptibility or resistance of cancer cells to apoptosis-induced cell death, thereby influencing both pro-cancer effects and resistance towards chemotherapy treatment (Wang X. et al., 2022; Zhang et al., 2022). It is intricately associated with the proliferation, differentiation, apoptosis, vasculogenesis, migration, and invasion of cancer cells (Ciaglia et al., 2023). It has been shown that the use of agonists and antagonists can modulate the TRP channel activity in diverse cancer models, suggesting that they are good potential therapeutic targets (Koivisto et al., 2022). However, The majority of these agonists or antagonists lack specificity and only impact a limited number of TRP channels (Ciaglia et al., 2023). TRP channels are widely present in other tissues in the body, and these agonists or antagonists can affect their involvement in various tissues, leading to negative effects (Santoni et al., 2019; Saldías et al., 2021; Sakellakis et al., 2024; Stokłosa et al., 2020). This is by far the biggest limitation.

The expression levels of various TRP channels in different types of cancers have been extensively studied, but there are a limited number of studies investigating the changes in TRP channel levels during cancer development and pathophysiological processes (Abid et al., 2024; Santoni et al., 2019; Saldías et al., 2021). By exploring this pathway, the relationship between changes in the expression of TRPs in patients and cancer can be further clarified, as well as the precise treatment for the alterations in the expression levels of various TRP channels and mutations in TRPs, which is expected to improve the survival rate of cancer patients as well as to reduce the adverse effects during the treatment process (Fraser et al., 2005).

## 3 Role of TRPs in breast cancer development and pathophysiological processes

Mutations in genes encoding TRP channels lead to dysregulation of TRP channels expression and disrupt the typical spatiotemporal pattern of local intracellular Ca^2+^ distribution (Hu et al., 2023). Dysregulation of multiple downstream Ca^2+^ homeostasis-dependent effectors has been linked to characteristics of breast cancer pathogenesis, including enhanced proliferation, increased cell survival, and augmented invasive potential in breast cancer cells. The involvement of TRP channels in promoting cellular proliferation, aberrant differentiation, and apoptotic damage contributes to uncontrolled expansion and invasive growth of breast cancer (Table 2). TRP channels exert significant influence on multiple cellular events governing cell fate determination and play a pivotal role in the progression of breast cancer (Prevarskaya et al., 2007; Gkika and Prevarskaya, 2009; Santoni et al., 2011).

**TABLE 2:** Role of TRPs in breast cancer development and pathophysiological processes.

Regulatory mechanisms	TRPC	TRPV	TRPP	TRPA	TRPML	TRPM	Reference
proliferation	TRPC1 TRPC3	TRPV1 TRPV2 TRPV6				TRPM2 TRPM7 TRPM8	Davis et al. (2012), Hiani et al. (2006), Hiani et al. (2009a), Hiani et al. (2009b), Faouzi et al. (2016), Riehle et al. (2018), Vercelli et al. (2015), Weber et al. (2016), Zheng et al. (2016), Dhingra et al. (1995), TaiYK et al. (2022), Santoni et al. (2020), Elbaz et al. (2016), Chakrabarti et al. (2009), Peters et al. (2012), Xu et al. (2021), Pan et al. (2013), Hopkins et al. (2015), Koh et al., 2015; Miller (2019), Dhennin-Duthille et al. (2011), Guilbert et al. (2009), Song et al. (2017)
Apoptosis	TRPC1 TRPC3	TRPV1 TRPV4 TRPV6					Galluzzi et al. (2018), Zhang et al. (2020), Wang et al. (2019), Kitazumi and Tsukahara (2011), Chahl (2024), Munjuluri et al. (2021), Nur et al. (2017), Nazıroğlu et al. (2017), Koşar et al. (2016), Ramírez-Barrantes et al. (2018), Wu et al. (2014), Peters et al. (2017), Bolanz et al. (2008), Zheng et al. (2007)
Autophagy						TRPM2 TRPM8	Miller, 2019; Wang et al. (2016), Huang et al. (2020)
Metastasis	TRPC1 TRPC2	TRPV4 TRPV6				TRPM4 TRPM7 TRPM8	Guilbert et al. (2013), Middelbeek et al. (2012), Wang et al. (2022b), Kuipers et al. (2018), Shu et al. (2021), Davis et al. (2014), Rivas et al. (2020), Wong and Hussain (2020), Coticchia et al. (2009), Deb et al. (2004), Chodon et al. (2010), Liu et al. (2014), Yapa et al. (2018), Lee et al. (2016), Neuberger and Sobolevsky (2023), Xu et al. (2018), Nguyen et al. (2017), Yuan et al. (2009), Derler et al. (2013)
Angiogenesis	TRPC1 TRPC3 TRPC4 TRPC6	TRPV1 TRPV4		TRPA1			Khalil et al. (2018), Lehen’kyi and Prevarskaya (2011), Ching et al. (2011), Fiorio Pla et al. (2012), Ma et al. (2011), Ma et al. (2010)

### 3.1 Proliferation

The division and proliferation of normal cells is tightly regulated, and when it is not, cancer develops (Qiao et al., 2024). Almost all tumour cells have a characteristic in which genes involved in cell proliferation are switched on or activated, allowing the tumour to grow unchecked (Sánchez-Hernández et al., 2024). Biologically, drugs that induce tumour cell differentiation inhibit tumour cell proliferation or cause tumour cell death can exert anti-tumour effects. Therefore, exploring the relationship between TRP channels and breast cancer proliferation may provide a research basis for the development of new therapeutic targets.

#### 3.1.1 TRPC and proliferation

There are few studies on TRPC channels and breast cancer, but those that have been reported have targeted TRPC1 and TRPC3 as channels associated with breast cancer proliferation (Elzamzamy et al., 2020).

The role of TRPC1 for the proliferative aspect of breast cancer can be summarised as downregulation of TRPC1 expression reduces the rate of cellular proliferation. Knockdown of TRPC1 in MDA-MB-468 breast cancer cells attenuates unstimulated Ca^2+^ influx via ORAl1 in a Ca^2+^ influx-dependent manner, reduces cell proliferation rates and is associated with a decrease in S-phase cells (Davis et al., 2012). MCF-7 breast cancer cells proliferate when the Ca^2+^-sensing receptor (CaSR) is activated by extracellular Ca^2+^ or its agonist spermine (El Hiani et al., 2006; El Hiani et al., 2009a). Ca^2+^-mediated CaSR activation in MCF-7 cells leads to PLC and PKC activation (El Hiani et al., 2009b), while ERK1/2 is activated downstream of PLC and PKCMCF-7. Reduced TRPC1 expression inhibited CaSR activation-mediated ERK1/2 phosphorylation, which is required for CaSR-induced proliferation of MCF-7 cells. Therefore, reducing TRPC1 expression may inhibit MCF-7 cell proliferation through the above mechanism. Furthermore, in the study by Faouzi M et al. (2016), KCa3.1 regulates Ca^2+^ inward flow and cell proliferation in the MCF-7 cell model by functionally synergising with TRPC1 channels. Expression of both channels increased at the end of G1 phase, whereas silencing either KCa3.1 or TRPC1 decreased the number of cells proliferating, and inhibition of TRPC1 expression inhibited MCF-7 proliferation by halting cell cycle progression in G1 phase and thus inhibiting MCF-7 proliferation.

In a study on the mechanism of action of polyunsaturated fatty acids (PUFAs) on breast cancer, it was found that TRPC3 channels were highly expressed in MCF-7 breast cancer cells, and TRPC3 antagonists attenuated the proliferation of breast cancer cells, and that PUFAs directly inhibited TRPC3 in MCF-7 cells, which could be a potential target for the development of new anticancer drugs (Riehle et al., 2018).

#### 3.1.2 TRPV and proliferation

Vercelli et al. (2015) demonstrated inhibition of breast cancer cell proliferation using the TRPV1 agonist capsazepin. Interestingly, the TRPV1 inhibitor capsazepin showed the same results. In Lea V Weber’s (Weber et al., 2016) experiments, they also demonstrated a significant reduction in the growth rate of MCF-7 breast cancer cells after stimulation with the TRPV1 agonist capsaicin, a finding consistent with the results of Vercelli et al. Zheng et al. (2016) further showed that capsaicin was able to potentiate the antitumour effect of pirorubicin. Pirorubicin is an analogue of the antibiotic Adriamycin (Dhingra et al., 1995). The inhibitory effect of the TRPV1 signalling pathway on cell proliferation was also confirmed in HCT116 cells. In addition, TRPV1 was overexpressed in the triple-negative breast cancer (TNBC) and significantly inhibited cell proliferation (TaiYK et al., 2022).

TRPV2 is aberrantly expressed in almost all breast cancers, and TRPV2 also serves as a valuable biomarker and a promising candidate target for therapeutic intervention. TRPV2 plays an important regulatory role in breast cancer cells (Santoni et al., 2020). In invasive breast cancer cells, the TRPV2 agonist CBD inhibits ERK-dependent breast cancer proliferation through sustained upregulation of ERK activity (Santoni et al., 2020). Greater inhibition of breast tumour growth when TRPV2 channel activation is combined with chemotherapy (Elbaz et al., 2016). The overexpression of TRPV2 with CBD enhanced the susceptibility of triple-negative and estrogen beta-negative breast cancer cells to apoptosis induced by doxorubicin chemotherapy regimens. This serves as a positive prognostic factor (Chakrabarti et al., 2009; Pan et al., 2013). The proliferation of multiple human breast cancer cell lines is inhibited by trinilast (e.g., MDA-MB-231, BT-474, and MCF-7). In breast cancer cell line MCF-7, Trinilast blocks IGF-1-induced low TRPV2 expression by inhibiting voltage-independent Ca^2+^ channel-mediated calcium in-flow (Chakrabarti et al., 2009).

The dysregulation of TRPV6 levels, whether too low or too high, can disrupt the homeostasis of the mammary epithelium and potentially facilitate the progression of pathophysiological conditions. The TRPV 6-mediated Ca^2+^ internal circulation passes through the targeted IGF-mediated AKT-TOR and ERK signaling pathways to maintain the inactive state (Marini et al., 2023). TRPV6 channels are significantly expressed in breast cancer cells and are associated with malignancy and prognosis. Inhibiting TRPV6 channels reduces the proliferative capacity of breast cancer cells and enhances their sensitivity to chemotherapeutic agents (Peters et al., 2012; Xu et al., 2021). This may be related to the fact that TRPV6 promotes cell proliferation through the Ca^2+^-dependent calmodulin/calmodulin neurophosphatase/NFAT pathway. The activation of this pathway exerts an influence on genes implicated in cellular proliferation and matrix degradation through the mediation of MMPs (Pan et al., 2013). Targeted knockdown of TRPV6 in cells with elevated TRPV6 expression inhibits growth and cell cycle arrest, suggesting that targeted inhibition of TRPV6 channels might be a useful therapeutic mechanism (Peters et al., 2012; Xu et al., 2021). However, other intracellular pathways downstream of TRPV6 have been poorly studied and their significance for cancer progression also awaits further exploration (Kärki et al., 2020).

#### 3.1.3 TRPM and proliferation

TRPM2 is predominantly localized in the nuclear compartment of breast cancer cells, with approximately 40%–45% of TRPM2 residing within the nucleus and the remainder distributed among other subcellular portions such as the cytoplasm. The TRPM2 channel exhibited a pronounced protective effect in mitigating DNA damage in a human breast adenocarcinoma cell line. The pharmacological inhibition of TRPM2 through silencing its mRNA reduces cell proliferation and significantly enhances DNA damage (Hopkins et al., 2015). The inhibition of TRPM2 in triple-negative and estrogen receptor-positive breast cancer results in an augmentation of DNA damage and cytotoxicity (Koh et al., 2015). BarbaraA.Miller speculates that mechanisms associated with cytosolic TRPM2 channels include cytosolic TRPM2 promoting DNA repair or promoting nuclear calcium inward flow, but this needs to be explored further. High levels of oxidative stress found in TRPM2 deficient neuroblastoma cells may be a potential mechanism to explain the increased DNA damage found in breast cancer when TRPM2 is inhibited (Miller, 2019). In contrast, the localization of TRPM2 in non-cancerous MCF-10A mammary epithelial cells was not limited to the nucleus, and no impact on proliferation was observed upon inhibition of TRPM2 (Miller, 2019). Therefore, targeted TRPM2 therapy may be a neoadjuvant treatment that can improve the treatment of breast cancer patients, including chemotherapy-resistant patients.

Dhennin-Duthille et al. (2011) showed that human breast ductal adenocarcinoma (hBDA) tissues had higher expression levels of TRPM7 and TRPM8 compared to neighbouring non-tumour tissues. And the expression of TRPM7 and TRPM8 exhibited significant associations with the tumour Ki67 proliferation index, tumour size, and Scarff-Bloom-Richardson (SBR) grade. Ki67 mitotic markers positively correlate with upregulated TRPM7 channels in breast cancer tissues, suggesting a role for this channel in breast cancer cell proliferation. TRPM7 silencing also inhibited the proliferation of breast cancer cell lines *in vitro* (Guilbert et al., 2009). However, because the channel has the ability to permeate both Ca^2+^/Mg^2+^ and kinases, it is tentatively undetermined whether this effect is mediated solely by the Ca^2+^ signalling pathway. However, The TRPM7 kinase inhibitor TG100-115 exhibited little impact on the proliferation of MDA-MB-231 breast cancer cells, suggesting that the anti-proliferative effect of TRPM7 silencing is not likely tod be related to the structural domain of TRPM7 kinase (Song et al., 2017).

The respective manifestations of TRPM channels in breast cancer have been partially elucidated, but further investigation is needed to understand how their individual effects are modulated and whether there is any overlap or integration between their activities.

### 3.2 Apoptosis

Apoptosis is one of the main mechanisms of tumour therapy, which is cellular suicide resulting from the activation of energy-dependent death programmes within the cell, and is a genetically controlled process of autonomous and ordered active cell death (Galluzzi et al., 2018). Monitoring the apoptotic response after tumour treatment is important for assessing efficacy and optimising and managing treatment strategies. Many drugs inhibit tumour growth by promoting apoptosis. For example, the anti-tumour effects of EGFR inhibitors are mediated by remote control of BCL-2 family member expression and apoptosis. p53 directly regulates the transcription of death receptors such as Fas or DR5, and stabilisation of p53 by drugs can induce apoptosis by both endogenous and exogenous pathways.

Within the TRP family, several TRP channels have been shown to be inextricably linked to apoptosis in breast cancer cells.

#### 3.2.1 TRPC and apoptosis

In a study by Zhang et al. (2020), MCF-7 breast cancer cells were found to have an elevated rate of apoptosis when co-cultured with the TRPC1 inhibitor SKF-96365. TRPC3 expression was elevated in the triple-negative MDA-MB-231 cell line compared to MCF-7 cells, and its inhibitory effect significantly reduced cell proliferation and increased apoptosis (Wang et al., 2019). Caspase-3/7 cleaves DNA repair enzymes and inactivates their DNA repair capacity during apoptosis (Kitazumi and Tsukahara, 2011). Yan Wang et al. (Wang et al., 2019)found that blocking TRPC3 increased the amount of cleaved caspase-3/7, suggesting that blocking TRPC3 induces caspase-dependent apoptosis. At the same time they highlighted the critical functional role of the TRPC3-RASA4-MAPK signalling cascade in maintaining TNBC cell proliferation and apoptosis resistance.

#### 3.2.2 TRPV and apoptosis

The TRPV1 channels serve as specific molecular targets for vanilloid capsaicinoids, while they can also be activated by acids (pH < 6.5), ethanol, and heat (Chahl, 2024). The TRPV1 receptor is predominantly located in the plasma membrane, with additional presence detected in the sarcoplasmic reticulum and sarcoplasmic reticulum, thereby facilitating intracellular release of Ca^2+^ from these compartments (Munjuluri et al., 2021). TRPV1 activation has been linked by several studies to an anti-tumour effect in breast cancer, where it promotes cell death and may reduce cell proliferation, and TRPV1 channel agonists may have a therapeutic effect (Nur et al., 2017; Nazıroğlu et al., 2017). TRPV1, in conjunction with agonists, modulators (such as MRS1477), and chemotherapeutic agents like cisplatin, triggers apoptosis through mitochondrial membrane depolarization, generation of reactive oxygen species (ROS), and activation of caspases (Nur et al., 2017; Nazıroğlu et al., 2017). Similarly, the combination of melatonin and the chemotherapeutic drug DOX induces apoptosis in MCF-7 cells by activating TRPV1 (Koşar et al., 2016). Furthermore, it was observed that low doses of capsaicin triggered apoptosis in tumor cells through TRPV1 activation, while high doses of capsaicin resulted in necrosis (Ramírez-Barrantes et al., 2018). A study by Wu et al. (2014) showed that necrosis of MCF-7 cells after treatment with the TRPV1 agonist capsaicin was dependent on TRPV1 induction. Therefore, the utilization of TRPV1 agonists could be a complementary approach to current therapeutic agents to promote apoptosis in drug-resistant tumour cells.

In basal breast cancer TRPV4 is overexpressed, and MDA-MB-468 breast cancer cells which expresses high levels of TRPV4 induce death by both necrosis and apoptosis in response to activation of TRPV4 by GSK1016790A (Peters et al., 2017). In contrast, TRPV4 silencing reduced the above effect, which was not observed in breast cancer cell lines exhibiting low or negligible levels of TRPV4 expression (Peters et al., 2017), thereby suggesting that the mechanism underlying GSK1016790A-induced cell death is mediated through TRPV4.

The TRPV6 channel may also also represent a promising therapeutic target, and its elevated expression in breast cancer is associated with reduced survival rates (Peters et al., 2012). The knockdown of TRPV6 resulted in a reduction in proliferation and an increase in apoptosis in T47D cells (Bolanz et al., 2008). By caspase-3/caspase-7 assay, KatrinA et al. (Bolanz et al., 2008)found a slight increase in breast cancer cell apoptosis after treatment with TRPV6-specific siRNAs. The activity of the oestrogen receptor modulator tamoxifen works better in breast tumours expressing oestrogen receptor and/or progesterone receptor by blocking oestrogen signalling through the oestrogen receptor and reducing growth signals to the cells. In a study by KatrinA et al. (Bolanz et al., 2008), the mRNA expression of TRPV6 was found to be upregulated by 2–15-fold compared to the average level in normal breast tissue. Additionally, the estrogen receptor antagonist tamoxifen exhibited a reduction in TRPV6 expression and demonstrated an enhancement of tamoxifen activity through inhibition of TRPV6 expression. High-dose tamoxifen (>5 Amol/L) induces apoptosis in T47D cells (Zheng et al., 2007). And they can affect channel activity more effectively by designing chemical inhibitors specific to TRPV6 (Bolanz et al., 2008). These studies suggest that combination therapy involving tamoxifen and TRPV6 inhibitors would be a promising approach for the treatment of breast cancer cells.

### 3.3 Autophagy

Autophagy is one of the pathways used to eliminate the cause of disease and phagocytosis of apoptotic cells (Miller, 2019). It plays a complex role in cancer, both in preventing bioenergetic failure due to metabolic stress, in controlling the quality and quantityof proteins andorganelles, and in supporting tumour development and maintaining the malignant state (Zhang et al., 2024). Autophagy inhibits tumourigenesis in the early stages oftumours due to a combination of unilateral or multiple effects of tumour microenvironment (TME) stress, pathogenic conditions and the immune system; however, in the advanced stages of tumours autophagy promotes tumourigenesis and increases tumour cell growth and metastasis (Zhang et al., 2024). As a result, autophagy has become a popular target for anti-cancer therapy.

TRPM2 has been shown to regulate autophagy in neuroblastoma, gastric cancer, breast cancer and others through several pathways (Miller, 2019). In HeLa cells, oxidative stress-induced TRPM2-mediated Ca^2+^ in-flow leads to phosphorylation and activation of calcium/calmodulin-dependent protein kinase II (CAMKII), followed by CAMKII phosphorylation of BECN1/BeclinBECN1 dissociates from PIK3C3 and binds Bcl-2 to inhibit autophagy, making the cells more susceptible to death (Wang et al., 2016)^.^

Yuan Huang et al. (2020) showed that TRPM8 serves as a universal master regulator of autophagy, maintaining and regulating autophagy levels across various types of mammalian cancer cells. Furthermore, TRPM8 is dependent on proliferation for autophagy and migration of breast cancer cells. Since the upregulation of TRPM8 channels has been linked to various autophagy-related cancers, suggesting that targeting the regulation of autophagy through TRPM8 channels could be a potential therapeutic strategy (Wang et al., 2016).

### 3.4 Metastasis

Some components within the TRP channel superfamily exhibit functional associations with cellular events and structures that undergo mechanical transduction during cell migration, including the actin cytoskeleton and focal adhesion (Hu et al., 2023). Thus, the involvement of these factors in mechanotransduction significantly enhances metastasis, potentially through robustly promoting the migration and invasion of cancer cells.

#### 3.4.1 TRPM and metastasis

The TRPM channels play a crucial role in modulating breast cancer metastasis, and the role of TRPM7 in particular in influencing metastasis in cancer has been explored in greater depth. The de-adhesion and myosin II-based cell-substrate interactions in MDA-MB-213 breast cancer cells are found to be TRPM7-dependent. And in a mouse breast cancer model, TRPM7 is required for cell metastasis to the lungs (Guilbert et al., 2013). A study utilizing a xenograft model of human breast cancer in mice demonstrated the key role of TRPM7 in tumor metastasis. Elevated mRNA expression of TRPM7 was found to significantly shorten the recurrence-free survival period among patients with primary breast cancer (Middelbeek et al., 2012). Moreover, targeted silencing of TRPM7 in MDA-MB-435 or MDA-MB-231 breast cancer cells through gene knockout resulted in enhanced contractility and increased focal adhesions, which were closely associated with diminished migratory and invasive capabilities (Middelbeek et al., 2012).

Regarding the possible mechanism of TRPM 7 with metastasis, direct phosphorylation of the structural domain myosin heavy chain (MHC) of TRPM7 kinase was found to be confirmed in MDA- MB-231 cells. Also overexpression of TRPM7 increased migration of MCF-7 and MDA-MB-213 cells, whereas a truncated version lacking the structural domain of the kinase had no effect on cell migration (Guilbert et al., 2013). The silencing of TRPM7 in MDA-MB-231 cells leads to a reorganization of the actin cytoskeleton and enhances cellular contraction (Middelbeek et al., 2012). These results suggest that TRPM7 may regulate cell migration and promote infiltration and metastasis in breast cancer by phosphorylating MHC. Therefore, repressing the connection between TRPM7 and MHC, along with impeding its phosphorylation, may present a promising therapeutic approach for diminishing the occurrence of breast cancer metastases (Saldías et al., 2021). Furthermore, the methylation of TRPM7 showed a negative correlation with lymph node metastasis, disease recurrence and ultimately cancer-induced mortality (Wang X. et al., 2022). Moreover, the TRPM7 channels exert a cytoskeletal stress-reducing effect by inhibiting the activity of myosin II, which mechanistically triggers the activation of SOX4 expression and thus contributes to the metastatic process in breast cancer cells (Kuipers et al., 2018). These results suggest that TRPM7 channels are involved in altered mechanical adhesion dynamics and reduced cytoskeletal tension in breast cancer, which ultimately leads to increased cell migration (Kuipers et al., 2018).

The tumor microenvironment can induce cancer cells to undergo epithelial-mesenchymal transition (EMT), thereby promoting the acquisition of an aggressive phenotype (Shu et al., 2021). Davis FM et al. demonstrated that the stimulus employed to induce epithelial-mesenchymal transition (EMT) elicits a transient elevation in cytosolic calcium levels within human breast cancer cells. Their findings revealed that the expression of the transient receptor potential-melastatin like 7 (TRPM7) channel regulates EGF-induced phosphorylation of STAT3 and expression of the EMT-associated marker vimentin (Davis et al., 2014). The findings suggest that TRPM7 plays a partial role in regulating EMT in breast cancer cells, while other calcium-permeable ion channels are also implicated in the induction of calcium-dependent EMT. The intracellular calcium signaling plays a crucial role in inducing EMT in human breast cancer cells. Therefore, targeting the EMT-induced calcium signaling pathway could be a promising therapeutic approach to prevent metastasis.

“The upregulation of TRPM4 was observed in ER+/pr+ and the specimens of triple-negative breast cancer, as compared to normal breast tissue.” (Rivas et al., 2020; Wong and Hussain, 2020). In addition, according to TNM classification, The expression of TRPM4 was elevated in stage IIB, IIIA, and IV, also in breast cancers with lymph node spread (N1-N2) (Rivas et al., 2020; Wong and Hussain, 2020). The identification of TRPM4 interactions in mechanistic studies may provide valuable insights into the biological role of TRPM4 in breast cancer and facilitate the exploration of molecular targets for the treatment of breast cancer. The potassium channel tetramerization domain-containing protein 5 (KCTD5) interacts with TRPM4, acting as a positive regulator of TRPM4 activity and playing a regulatory role in cell migration and contraction. The expression of KCTD5 mRNA was significantly increased in breast cancer tumors (Rivas et al., 2020)^.^ . To sum up, increased KCTD5 expression may be a key factor in the metastasis of these types of tumours, which promotes TRPM4-dependent cell migration through its interaction with TRPM4 and enhancement of channel activity. In addition, The presence of CaM serves as another interacting factor that positively regulates channel activity. (Coticchia et al., 2009; Deb et al., 2004). TRPM4-CaM interactions in breast cancer can promote migration and shrinkage of tumour cells by increasing activity levels and channel sensitivity.

The expression of TRPM8 is upregulated in grade I adenocarcinomas of ER + breast cancer (Dhennin-Duthille et al., 2011; Chodon et al., 2010). The mRNA expression of TRPM8 was relatively high in the MDA-MB-231 cell line, renowned for its high aggressiveness. TRPM8 silencing reduces migration and invasion in MDA-MB-231 breast cancer cells. Enhanced invasion and migration when TRPM8 is overexpressed in a low invasive MCF-7 cell line (Dhennin-Duthille et al., 2011). In these cells, the activity of TRPM8 may be hormone-dependent, as its expression is regulated by estrogen receptor alpha (ERa) and estrogen, and is associated with the status of tumor estrogen receptors (Dhennin-Duthille et al., 2011; Chodon et al., 2010; Liu et al., 2014). TRPM8 regulates the process of epithelial-mesenchymal transition (EMT) by activating the AKT/GSK-3β pathway, thereby promoting metastasis in MCF-7 cells or MDA-MB-231 (Saldías et al., 2021; Liu et al., 2014). However, it has also been demonstrated that TRPM8 is not expressed in MDA-MB-231 cells, and the absence of TRPM8 transcripts was observed in half of the breast cancer cell lines examined. Thus the therapeutic potential of TRPM8 is very constrained (Yapa et al., 2018), but its elevated correlates can suggest a high risk of tumour metastasis.

#### 3.4.2 TRPV and metastasis

The expression of TRPV4 in human clinical samples, as observed in public databases, demonstrates an upregulation specifically in the underlying subtype of breast cancer. This increased expression is closely linked to a more aggressive phenotype and poorer survival outcomes. The novel role of TRPV4 in breast cancer metastasis was illustrated in a study by Lee et al. (2016). Both TRPV4 silencing and pharmacological inhibition of TRPV4 inhibited migration and invasion of the 4T07 breast cancer cell line expressing TRPV4, and 4α-PDD (a TRPV4 agonist) downregulated adherence-associated tumour suppressor genes in the 4T07 cells, further suggesting that TRPV4 increased the metastatic potential of the tumour (Lee et al., 2016). Although research on the potential role of TRPV4 in cancer metastasis is still in its early stages, the current research foundation could pave the way for further investigations into the involvement of TRPV4 in cancer pathogenesis. Additionally, there exists a significant possibility that targeting TRPV4 channels may hold therapeutic promise for treating metastatic cancers.

In addition, the stimulation of TRPV3 has the potential to trigger the expression of genes linked to the progression and metastasis of cancer, such as matrix metalloproteinases (MMPs) and vascular endothelial growth factor (VEGF) (Neuberger and Sobolevsky, 2023).

The function of TRPV6 overexpression in breast cancer proliferation has been described above, and it may also affect breast cancer prognosis by promoting breast cancer invasion and metastasis. Overexpression of TRPV6 in breast cancer is a common event, with 93.3% of biopsies showing higher levels of the protein than non-invasive tumour areas, particularly in invasive areas (Xu et al., 2018).

Enhanced expression of the TRPV6 calcium channel is associated with unfavorable outcomes in breast cancer due to its promotion of invasion and metastasis, indicating that targeting TRPV6 could be a promising strategy for breast cancer treatment. However, the mechanism by which TRPV6 promotes breast metastasis is unknown. Xu et al. (2021) reported that TRPV6 expression was upregulated in metastatic breast cancer and that overexpression or upregulation of TRPV6 accelerated the migration of primary breast cancer cells. Consistent with these findings, inhibition of TRPV6 reduced cell migration. Mechanistically, TRPV6 can activate NFATc2 by increasing the phosphorylation of nuclear factors in the nuclear factor of activated T cells two interacting protein (NFATc2IP), suggesting CDK5 as a potential candidate gene responsible for this phosphorylation. Thus, the activation of NFATc2 promotes breast cancer metastasis by upregulation of ADAM metalopeptidase with thrombospondin type 1 motif 6 (ADAMTS6) expression. The findings suggest that TRPV6 activation enhances NFATc2’s transcriptional activity by modulating the phosphorylation of NFATc2IP, thereby promoting ADAMTS6 expression and facilitating metastasis in breast cancer.

#### 3.4.3 TRPC and metastasis

TRPC channels have not been well studied in breast cancer metastasis, and TRPC2 silencing reduced detectable breast cancer lung metastases in a 4T1 mouse model of homozygous metastatic breast cancer (Nguyen et al., 2017). In contrast, the role of TRPC1 in breast cancer metastasis is inconsistent with TRPC2. TRPC1 is also highly expressed in breast cancer, and TRPC1 overexpression inhibits the proliferation and migration of ER-positive breast cancers. Interestingly, TRPC1 expression levels were completely different in different breast cancer cell lines (Yuan et al., 2009; Derler et al., 2013). A study by Zhang et al. (2020) found that TRPC1 overexpression in MCF-7 breast cancer cells reduced migration capacity by 64.8% (P = 0.002) and invasion capacity by 54.3% (P = 0.003). And SKF-96365, a TRPC1 inhibitor that inhibits TRPC1 expression, reversed MCF-7 migration and invasion caused by TRPC1 overexpression.

### 3.5 Angiogenesis

The internal environment in which tumour cells arise and live is the tumour microenvironment (TME), which includes tumour cells and their surrounding fibroblasts, immune and inflammatory cells, and glial cells, as well as the intercellular stroma, microvasculature, and biomolecules infiltrating them. The three features of hypoxia, chronic inflammation and immunosuppression form a complex network of mechanisms that play an important role in the development of tumours. Some TRP channels are expressed in immune cells, smooth muscle cells, vascular endothelial cells, stromal cells near the tumour, and sensory nerve endings (Khalil et al., 2018; Lehen’kyi and Prevarskaya, 2011). The modulation of sensory-vascular-immune-tumour interactions by these channels can exert influence on the tumour microenvironment (Nilius and Owsianik, 2011). Thus, the tumour microenvironment is one of the more promising therapeutic targets.

The process of neoangiogenesis is crucial for the growth of tumors, and TRP channels such as TRPC3, TRPC6, TRPV1, TRPV4, and TRPM4 play a regulatory role in vascular permeability and cancer vascular angiogenesis (Khalil et al., 2018). The tumour’s need for oxygen and nutrients allows the formation of tumour blood vessels, which through angiogenesis are key events in tumour progression (Lehen’kyi and Prevarskaya, 2011). The initiation of neovascularization in tumor-associated angiogenesis occurs within a hypoxic microenvironment, primarily attributed to the secretion of growth factors that can sensitize various TRP channels through tyrosine kinase-linked receptors.

There are fewer referable studies on TRP channels and tumour angiogenesis, and current relevant studies focus more on the relationship between TRP channel expression and endothelial cell biological behaviour in tumour angiogenesis. In an *in vivo* matrix plugging angiogenesis assay, a highly selective TRPV1 receptor agonist induced induced angiogenesis in wild-type mice, whereas TRPV1 knockout mice did not (Ching et al., 2011). Endothelial cells derived from breast cancer vessels contain high levels of TRPV4, and inhibition of TRPV4 inhibits the stimulated migration of endothelial cells derived from breast cancer, suggesting that pharmacological inhibition of TRPV4 can inhibit angiogenesis (Fiorio Pla et al., 2012). Furthermore, functional interactions between TRPC1 and TRPV4 are involved in endothelial cell Ca^2+^ homeostasis (Ma et al., 2011; Ma et al., 2010). The co-regulation of TRPC1 and TRPV4 plays a crucial role in tumor angiogenesis, involving Caveolin-1 and IP3R3. Further investigation is required to explore the functional analyses of these interactions and their cellular outcomes, aiming to utilize them as potential therapeutic targets (Saldías et al., 2021). The involvement of TRPA1 is also likely in this context, and its activation in prostate tumor endothelial cells serves as a regulatory factor for angiogenesis. Specifically, TRPA1 activation promotes neovascularization, migration of endothelial cells, and tubulogenesis within an *in vitro* model of human prostate cancer. It is therefore hypothesised that it may also be able to play a similar role in breast cancer. Angiogenesis plays a key role in tumour progression, so it is crucial to explore the relationship between TRP channels and angiogenesis.

## 4 Discussion

This paper reviews the important role played by TRP channels in various events of tumour development. Currently, more studies have focused on the TRPV, TRPC and TRPM families, which are closely related to tumour proliferation, apoptosis, metastasis, autophagy and angiogenesis. While some families of TRP channels and breast cancer have been poorly studied, others such as the TRPA family have studies on cancer pain and could also be a target for cancer pain relief. The present study will explore the link between TRP channels and breast cancer development, clarify their expression in breast cancer and summarise the regulation of these interactions as therapeutic targets. The resulting knowledge will provide new ideas for designing more specific anti-cancer drugs. As discussed herein, the role of TRPC, TRPV, and TRPM in breast cancer proliferation and metastasis, the role of TRPC and TRPV in breast cancer apoptosis, and the role of TRPM in breast cancer cell autophagy. As demonstrated in the aforementioned summary, the experiment demonstrated that the growth rate of MCF-7 breast cancer cells stimulated by the TRPV1 agonist capsaicin was significantly reduced. Consequently, it can be deduced that ‘activation of TRPV1 can inhibit breast cancer cell proliferation’, and the conclusion that ‘further development and application of TRPV1 agonists can be anti-tumour in terms of inhibiting proliferation’. Consequently, the hypothesis that ‘further development and application of TRPV1 agonists can be anti-tumour in terms of inhibiting proliferation’ can be formulated. This is predicated on the premise of developing drugs and applying the existing TRP channels, as well as conducting more in-depth research into the relationship between TRP channels and their upstream and downstream signalling pathways, in order to explore their specific mechanisms. In addition, altered expression of TRPs can be used as a predictive and diagnostic indicator of tumour development and prognosis.

However, particular attention needs to be paid during their drug discovery and development because inhibition of TRPs may lead to systemic toxicity due to their wide tissue distribution and multiple functions in the same types of cancers and healthy tissues. Thus, despite deriving a relationship between each TRP channel and tumours, this is still only the first step in the fight against tumours. The precise identification of distinct TRP channels and their accurate cellular localization in relation to specific cancer-promoting functions are crucial for the development of safer and more effective anti-cancer therapeutics. Further dedicated research is still needed to gain deeper insights into tumour biology powered by TRP channels.

## References

[B1] Abid A. Saeed H. Iftikhar U. Arshad M. K. Shahid M. U. Rasool T. (2024). A comprehensive study of adverse effects of chemotherapy on female breast cancer patients in NORI Cancer Hospital, Islamabad in a developing country. J. Oncol. Pharm. Pract., 10781552241266254. Epub ahead of print. 10.1177/10781552241266254 39090979

[B2] Bhaskaran M. D. Smith B. N. (2010). Effects of TRPV1 activation on synaptic excitation in the dentate gyrus of a mouse model of temporal lobe epilepsy. Exp. Neurol. 223 (2), 529–536. 10.1016/j.expneurol.2010.01.021 20144892 PMC2864369

[B3] Bolanz K. A. Hediger M. A. Landowski C. P. (2008). The role of TRPV6 in breast carcinogenesis. Mol. Cancer Ther. 7 (2), 271–279. 10.1158/1535-7163.MCT-07-0478 18245667

[B4] Cammann C. Kulla J. Wiebusch L. Walz C. Zhao F. Lowinus T. (2023). Proteasome inhibition potentiates Kv1.3 potassium channel expression as therapeutic target in drug-sensitive and -resistant human melanoma cells. Biomed. Pharmacother. 168, 115635. 10.1016/j.biopha.2023.115635 37816303

[B5] Chahl L. A. (2024). TRPV1 channels in the central nervous system as drug targets. Pharm. (Basel) 17 (6), 756. 10.3390/ph17060756 PMC11206835 38931423

[B6] Chakrabarti R. Subramaniam V. Abdalla S. Jothy S. Prud'homme G. J. (2009). Tranilast inhibits the growth and metastasis of mammary carcinoma. Anticancer Drugs 20 (5), 334–345. 10.1097/CAD.0b013e328327994e 19322072

[B7] Chen W. Li H. Hao X. Liu C. (2022b). TRPV1 in dorsal root ganglion contributed to bone cancer pain. Front. Pain Res. (Lausanne) 3, 1022022. 10.3389/fpain.2022.1022022 36438444 PMC9682177

[B8] Chen X. Wang M. Yu K. Xu S. Qiu P. Lyu Z. (2022a). Chronic stress-induced immune dysregulation in breast cancer: implications of psychosocial factors. J. Transl. Int. Med. 11 (3), 226–233. 10.2478/jtim-2021-0050 37662890 PMC10474889

[B9] Chen Z. Zhao Y. Tian Y. Cao R. Shang D. (2023). Pan-cancer analysis of the TRP family, especially TRPV4 and TRPC4, and its expression correlated with prognosis, tumor microenvironment, and treatment sensitivity. Biomolecules 13 (2), 282. 10.3390/biom13020282 36830651 PMC9953180

[B10] Ching L. C. Kou Y. R. Shyue S. K. Su K. H. Wei J. Cheng L. C. (2011). Molecular mechanisms of activation of endothelial nitric oxide synthase mediated by transient receptor potential vanilloid type 1. Cardiovasc Res. 91 (3), 492–501. 10.1093/cvr/cvr104 21493704

[B11] Chodon D. Guilbert A. Dhennin-Duthille I. Gautier M. Telliez M. S. Sevestre H. (2010). Estrogen regulation of TRPM8 expression in breast cancer cells. BMC Cancer 10, 212. 10.1186/1471-2407-10-212 20482834 PMC2887400

[B12] Ciaglia T. Vestuto V. Bertamino A. González-Muñiz R. Gómez-Monterrey I. (2023). On the modulation of TRPM channels: current perspectives and anticancer therapeutic implications. Front. Oncol. 12, 1065935. 10.3389/fonc.2022.1065935 36844925 PMC9948629

[B13] Coticchia C. M. Revankar C. M. Deb T. B. Dickson R. B. Johnson M. D. (2009). Calmodulin modulates Akt activity in human breast cancer cell lines. Breast Cancer Res. Treat. 115 (3), 545–560. 10.1007/s10549-008-0097-z 18587642 PMC3736740

[B14] Das R. Goswami C. (2019). TRPV4 expresses in bone cell lineages and TRPV4-R616Q mutant causing Brachyolmia in human reveals “loss-of-interaction” with cholesterol. Biochem. Biophys. Res. Commun. 517 (4), 566–574. 10.1016/j.bbrc.2019.07.042 31387748

[B15] Davis F. M. Azimi I. Faville R. A. Peters A. A. Jalink K. Putney J. W. Jr (2014). Induction of epithelial-mesenchymal transition (EMT) in breast cancer cells is calcium signal dependent. Oncogene 33 (18), 2307–2316. 10.1038/onc.2013.187 23686305 PMC3917976

[B16] Davis F. M. Peters A. A. Grice D. M. Cabot P. J. Parat M. O. Roberts-Thomson S. J. (2012). Non-stimulated, agonist-stimulated and store-operated Ca^2+^ influx in MDA-MB-468 breast cancer cells and the effect of EGF-induced EMT on calcium entry. PLoS One 7 (5), e36923. 10.1371/journal.pone.0036923 22666335 PMC3364242

[B17] Deb T. B. Coticchia C. M. Dickson R. B. (2004). Calmodulin-mediated activation of Akt regulates survival of c-Myc-overexpressing mouse mammary carcinoma cells. J. Biol. Chem. 279 (37), 38903–38911. 10.1074/jbc.M405314200 15247222

[B18] Derler I. Plenk P. Fahrner M. Muik M. Jardin I. Schindl R. (2013). The extended transmembrane Orai1 N-terminal (ETON) region combines binding interface and gate for Orai1 activation by STIM1. J. Biol. Chem. 288 (40), 29025–29034. 10.1074/jbc.M113.501510 23943619 PMC3790000

[B19] Dhennin-Duthille I. Gautier M. Faouzi M. Guilbert A. Brevet M. Vaudry D. (2011). High expression of transient receptor potential channels in human breast cancer epithelial cells and tissues: correlation with pathological parameters. Cell Physiol. Biochem. 28 (5), 813–822. 10.1159/000335795 22178934

[B20] Dhingra K. Frye D. Newman R. A. Walters R. Theriault R. Fraschini G. (1995). Phase II clinical and pharmacological study of pirarubicin in combination with 5-fluorouracil and cyclophosphamide in metastatic breast cancer. Clin. Cancer Res. 1 (7), 691–697. 9816034

[B21] Elbaz M. Ahirwar D. Xiaoli Z. Zhou X. Lustberg M. Nasser M. W. (2016). TRPV2 is a novel biomarker and therapeutic target in triple negative breast cancer. Oncotarget 9 (71), 33459–33470. 10.18632/oncotarget.9663 30323891 PMC6173360

[B22] El Hiani Y. Ahidouch A. Lehen'kyi V. Hague F. Gouilleux F. Mentaverri R. (2009b). Extracellular signal-regulated kinases 1 and 2 and TRPC1 channels are required for calcium-sensing receptor-stimulated MCF-7 breast cancer cell proliferation. Cell Physiol. Biochem. 23 (4-6), 335–346. 10.1159/000218179 19471101

[B23] El Hiani Y. Ahidouch A. Roudbaraki M. Guenin S. Brûlé G. Ouadid-Ahidouch H. (2006). Calcium-sensing receptor stimulation induces nonselective cation channel activation in breast cancer cells. J. Membr. Biol. 211 (2), 127–137. 10.1007/s00232-006-0017-2 17041782

[B24] El Hiani Y. Lehen'kyi V. Ouadid-Ahidouch H. Ahidouch A. (2009a). Activation of the calcium-sensing receptor by high calcium induced breast cancer cell proliferation and TRPC1 cation channel over-expression potentially through EGFR pathways. Arch. Biochem. Biophys. 486 (1), 58–63. 10.1016/j.abb.2009.03.010 19332022

[B25] Elzamzamy O. M. Penner R. Hazlehurst L. A. (2020). The role of TRPC1 in modulating cancer progression. Cells 9 (2), 388. 10.3390/cells9020388 32046188 PMC7072717

[B26] Faouzi M. Hague F. Geerts D. Ay A. S. Potier-Cartereau M. Ahidouch A. (2016). Functional cooperation between KCa3. 1 and TRPC1 channels in human breast cancer: role in cell proliferation and patient prognosis. Oncotarget 7 (24), 36419–36435. 10.18632/oncotarget.9261 27183905 PMC5095010

[B27] Fiorio Pla A. Ong H. L. Cheng K. T. Brossa A. Bussolati B. Lockwich T. (2012). TRPV4 mediates tumor-derived endothelial cell migration via arachidonic acid-activated actin remodeling. Oncogene 31 (2), 200–212. 10.1038/onc.2011.231 21685934 PMC5934994

[B28] Foster H. M. Carle M. N. Jira L. R. Koh D. W. (2023). TRPM2 channels: a potential therapeutic target in melanoma? Int. J. Mol. Sci. 24 (13), 10437. 10.3390/ijms241310437 37445615 PMC10341991

[B29] Fraser S. P. Diss J. K. ChioniAM M. M. E. Pan H. Yamaci R. F. Pani F. (2005). Voltage-gated sodium channel expression and potentiation of human breast cancer metastasis. Clin. Cancer Res. 11 (15), 5381–5389. 10.1158/1078-0432.CCR-05-0327 16061851

[B30] Galluzzi L. Vitale I. Aaronson S. A. Abrams J. M. Adam D. Agostinis P. (2018). Molecular mechanisms of cell death: recommendations of the nomenclature committee on cell death 2018. Cell Death Differ. 25 (3), 486–541. 10.1038/s41418-017-0012-4 29362479 PMC5864239

[B31] Gkika D. Prevarskaya N. (2009). Molecular mechanisms of TRP regulation in tumor growth and metastasis. Biochim. Biophys. Acta 1793 (6), 953–958. 10.1016/j.bbamcr.2008.11.010 19103233

[B32] Guilbert A. Gautier M. Dhennin-Duthille I. Haren N. Sevestre H. Ouadid-Ahidouch H. (2009). Evidence that TRPM7 is required for breast cancer cell proliferation. Am. J. Physiol. Cell Physiol. 297 (3), C493–C502. 10.1152/ajpcell.00624.2008 19515901

[B33] Guilbert A. Gautier M. Dhennin-Duthille I. Rybarczyk P. Sahni J. Sevestre H. (2013). Transient receptor potential melastatin 7 is involved in oestrogen receptor-negative metastatic breast cancer cells migration through its kinase domain. Eur. J. Cancer 49 (17), 3694–3707. 10.1016/j.ejca.2013.07.008 23910495

[B34] Guo W. Tang Q. Wei M. Kang Y. Wu J. X. Chen L. (2022). Structural mechanism of human TRPC3 and TRPC6 channel regulation by their intracellular calcium-binding sites. Neuron 110 (6), 1023–1035.e5. 10.1016/j.neuron.2021.12.023 35051376

[B35] Hopkins M. M. Feng X. Liu M. Parker L. P. Koh D. W. (2015). Inhibition of the transient receptor potential melastatin-2 channel causes increased DNA damage and decreased proliferation in breast adenocarcinoma cells. Int. J. Oncol. 46, 2267–2276. 10.3892/ijo.2015.2919 25760245 PMC4383028

[B36] Hu W. Wartmann T. Strecker M. Perrakis A. Croner R. Szallasi A. (2023). Transient receptor potential channels as predictive marker and potential indicator of chemoresistance in colon cancer. Oncol. Res. 32 (1), 227–239. 10.32604/or.2023.043053 38188686 PMC10767253

[B37] Huang Y. Li S. Jia Z. Zhao W. Zhou C. Zhang R. (2020). Transient receptor potential melastatin 8 (TRPM8) channel regulates proliferation and migration of breast cancer cells by activating the AMPK-ULK1 pathway to enhance basal autophagy. Front. Oncol. 10, 573127. 10.3389/fonc.2020.573127 33344232 PMC7746826

[B38] Kärki T. Rajakylä E. K. Acheva A. Tojkander S. (2020). TRPV6 calcium channel directs homeostasis of the mammary epithelial sheets and controls epithelial mesenchymal transition. Sci. Rep. 10 (1), 14683. 10.1038/s41598-020-71645-z 32895467 PMC7477193

[B39] Khalil M. Alliger K. Weidinger C. Yerinde C. Wirtz S. Becker C. (2018). Functional role of transient receptor potential channels in immune cells and epithelia. Front. Immunol. 9, 174. 10.3389/fimmu.2018.00174 29467763 PMC5808302

[B40] Kitazumi I. Tsukahara M. (2011). Regulation of DNA fragmentation: the role of caspases and phosphorylation. FEBS J. 278 (3), 427–441. 10.1111/j.1742-4658.2010.07975.x 21182594

[B41] Koh D. W. Powell D. P. Blake S. D. Hoffman J. L. Hopkins M. M. Feng X. (2015). Enhanced cytotoxicity in triple-negative and estrogen receptor-positive breast adenocarcinoma cells due to inhibition of the transient receptor potential melastatin-2 channel. Oncol. Rep. 34 (3), 1589–1598. 10.3892/or.2015.4131 26178079 PMC4735697

[B42] Koivisto A. P. Belvisi M. G. Gaudet R. Szallasi A. (2022). Advances in TRP channel drug discovery: from target validation to clinical studies. Nat. Rev. Drug Discov. 21 (1), 41–59. 10.1038/s41573-021-00268-4 34526696 PMC8442523

[B43] Koşar P. A. Nazıroğlu M. Övey İ. S. Çiğ B. (2016). Synergic effects of doxorubicin and melatonin on apoptosis and mitochondrial oxidative stress in MCF-7 breast cancer cells: involvement of TRPV1 channels. J. Membr. Biol. 249, 129–140. 10.1007/s00232-015-9855-0 26525975

[B44] Kuipers A. J. Middelbeek J. Vrenken K. Pérez-González C. Poelmans G. Klarenbeek J. (2018). TRPM7 controls mesenchymal features of breast cancer cells by tensional regulation of SOX4. Biochim. Biophys. Acta Mol. Basis Dis. 1864 (7), 2409–2419. 10.1016/j.bbadis.2018.04.017 29684587

[B45] Kumar B. Dreja K. Shah S. S. Cheong A. Xu S. Z. Sukumar P. (2006). Upregulated TRPC1 channel in vascular injury *in vivo* and its role in human neointimal hyperplasia. Circ. Res. 98 (4), 557–563. 10.1161/01.RES.0000204724.29685.db 16439693 PMC2633624

[B46] Kuwahara K. Wang Y. McAnally J. Richardson J. A. Bassel-Duby R. Hill J. A. (2006). TRPC6 fulfills a calcineurin signaling circuit during pathologic cardiac remodeling. J. Clin. Invest 116 (12), 3114–3126. 10.1172/JCI27702 17099778 PMC1635163

[B47] Landini L. Souza Monteiro de Araujo D. Titiz M. Geppetti P. Nassini R. De Logu F. (2022). TRPA1 role in inflammatory disorders: what is known so far? Int. J. Mol. Sci. 23 (9), 4529. 10.3390/ijms23094529 35562920 PMC9101260

[B48] Lee W. H. Choong L. Y. Mon N. N. Lu S. Lin Q. Pang B. (2016). TRPV4 regulates breast cancer cell extravasation, stiffness and actin cortex. Sci. Rep. 6, 27903. 10.1038/srep27903 27291497 PMC4904279

[B49] Lehen'kyi V. Prevarskaya N. (2011). Oncogenic TRP channels. Adv. Exp. Med. Biol. 704, 929–945. 10.1007/978-94-007-0265-3_48 21290334

[B50] Li Z. Zhang J. Ren X. Liu Q. Yang X. (2018). The mechanism of quercetin in regulating osteoclast activation and the PAR2/TRPV1 signaling pathway in the treatment of bone cancer pain. Int. J. Clin. Exp. Pathol. 11 (11), 5149–5156. 31949595 PMC6963045

[B51] Liu J. Chen Y. Shuai S. Ding D. Li R. Luo R. (2014). TRPM8 promotes aggressiveness of breast cancer cells by regulating EMT via activating AKT/GSK-3β pathway. Tumour Biol. 35 (9), 8969–8977. 10.1007/s13277-014-2077-8 24903376

[B52] Ma X. Cheng K. T. Wong C. O. O'Neil R. G. Birnbaumer L. Ambudkar I. S. (2011). Heteromeric TRPV4-C1 channels contribute to store-operated Ca^2+^ entry in vascular endothelial cells. Cell Calcium 50 (6), 502–509. 10.1016/j.ceca.2011.08.006 21930300 PMC3818799

[B53] Ma X. Qiu S. Luo J. Ma Y. Ngai C. Y. Shen B. (2010). Functional role of vanilloid transient receptor potential 4-canonical transient receptor potential 1 complex in flow-induced Ca2+ influx. Arterioscler. Thromb. Vasc. Biol. 30 (4), 851–858. 10.1161/ATVBAHA.109.196584 20093626

[B54] Marini M. Titiz M. Souza Monteiro de Araújo D. Geppetti P. Nassini R. De Logu F. (2023). TRP channels in cancer: signaling mechanisms and translational approaches. Biomolecules 13 (10), 1557. 10.3390/biom13101557 37892239 PMC10605459

[B55] Middelbeek J. Kuipers A. J. Henneman L. Visser D. Eidhof I. van Horssen R. (2012). TRPM7 is required for breast tumor cell metastasis. Cancer Res. 72 (16), 4250–4261. 10.1158/0008-5472.CAN-11-3863 22871386

[B56] Miller B. A. (2019). TRPM2 in cancer. Cell Calcium 80, 8–17. 10.1016/j.ceca.2019.03.002 30925291 PMC6545160

[B57] Morelli M. B. Amantini C. (2022). Transient receptor potential (TRP) channels: markers and therapeutic targets for cancer? Biomolecules 12 (4), 547. 10.3390/biom12040547 35454136 PMC9032313

[B58] Munjuluri S. Wilkerson D. A. Sooch G. Chen X. White F. A. Obukhov A. G. (2021). Capsaicin and TRPV1 channels in the cardiovascular system: the role of inflammation. Cells 11 (1), 18. 10.3390/cells11010018 35011580 PMC8750852

[B59] Nazıroğlu M. Çiğ B. Blum W. Vizler C. Buhala A. Marton A. (2017). Targeting breast cancer cells by MRS1477, a positive allosteric modulator of TRPV1 channels. PLoS One 12 (6), e0179950. 10.1371/journal.pone.0179950 28640864 PMC5481018

[B60] Neuberger A. Sobolevsky A. I. (2023). Molecular pharmacology of the onco-TRP channel TRPV6. Channels (Austin). 17(1). 10.1080/19336950.2023.2266669 PMC10578198 37838981

[B61] Nguyen O. N. Grimm C. Schneider L. S. Chao Y. K. Atzberger C. Bartel K. (2017). Two-pore channel function is crucial for the migration of invasive cancer cells. Cancer Res. 77 (6), 1427–1438. 10.1158/0008-5472.CAN-16-0852 28108508

[B62] Nilius B. Owsianik G. (2011). The transient receptor potential family of ion channels. Genome Biol. 12 (3), 218. 10.1186/gb-2011-12-3-218 21401968 PMC3129667

[B63] Nolan E. Lindeman G. J. Visvader J. E. (2023). Deciphering breast cancer: from biology to the clinic. Cell 186 (8), 1708–1728. 10.1016/j.cell.2023.01.040 36931265

[B64] Nur G. Nazıroğlu M. Deveci H. A. (2017). Synergic prooxidant, apoptotic and TRPV1 channel activator effects of alpha-lipoic acid and cisplatin in MCF-7 breast cancer cells. J. Recept Signal Transduct. Res. 37 (6), 569–577. 10.1080/10799893.2017.1369121 28849985

[B65] Owsianik G. Voets T. Peters J. A. (2007). Transient receptor potential cation channels in disease. Physiol. Rev. 87 (1), 165–217. 10.1152/physrev.00021.2006 17237345

[B66] Pan M. G. Xiong Y. Chen F. (2013). NFAT gene family in inflammation and cancer. Curr. Mol. Med. 13 (4), 543–554. 10.2174/1566524011313040007 22950383 PMC3694398

[B67] Payne S. L. Ram P. Srinivasan D. H. Le T. T. Levin M. Oudin M. J. (2022). Potassium channel-driven bioelectric signalling regulates metastasis in triple-negative breast cancer. EBioMedicine 75, 103767. 10.1016/j.ebiom.2021.103767 34933180 PMC8688589

[B68] Peters A. A. Jamaludin S. Y. N. Yapa KTDS Chalmers S. Wiegmans A. P. Lim H. F. (2017). Oncosis and apoptosis induction by activation of an overexpressed ion channel in breast cancer cells. Oncogene 36 (46), 6490–6500. 10.1038/onc.2017.234 28759041

[B69] Peters A. A. Simpson P. T. Bassett J. J. Lee J. M. Da Silva L. Reid L. E. (2012). Calcium channel TRPV6 as a potential therapeutic target in estrogen receptor-negative breast cancer. Mol. Cancer Ther. 11 (10), 2158–2168. 10.1158/1535-7163.MCT-11-0965 22807578

[B70] Prevarskaya N. Zhang L. Barritt G. (2007). TRP channels in cancer. Biochim. Biophys. Acta 1772 (8), 937–946. 10.1016/j.bbadis.2007.05.006 17616360

[B71] Qiao S. Wu F. Wang H. (2024). Genetic and immune identification and functional analysis of TRPM8 as a potential biomarker for pancreatic adenocarcinoma proliferation. Cancer Rep. Hob. 7 (6), e2108. 10.1002/cnr2.2108 PMC11150080 38837874

[B72] Ramírez-Barrantes R. Córdova C. Gatica S. Rodriguez B. Lozano C. Marchant I. (2018). Transient receptor potential vanilloid 1 expression mediates capsaicin-induced cell death. Front. Physiol. 9, 682. 10.3389/fphys.2018.00682 29922176 PMC5996173

[B73] Riehle M. Tsvetkov D. Gohlke B. O. Preissner R. Harteneck C. Gollasch M. (2018). Molecular basis for the sensitivity of TRP channels to polyunsaturated fatty acids. Naunyn Schmiedeb. Arch. Pharmacol. 391 (8), 833–846. 10.1007/s00210-018-1507-3 PMC6061713 29736621

[B74] Rivas J. Díaz N. Silva I. Morales D. Lavanderos B. Álvarez A. (2020). KCTD5, a novel TRPM4-regulatory protein required for cell migration as a new predictor for breast cancer prognosis. FASEB J. 34 (6), 7847–7865. 10.1096/fj.201901195RRR 32301552

[B75] Sakellakis M. Yoon S. M. Reet J. Chalkias A. (2024). Novel insights into voltage-gated ion channels: translational breakthroughs in medical oncology. Channels (Austin) 18 (1), 2297605. 10.1080/19336950.2023.2297605 38154047 PMC10761148

[B76] Saldías M. P. Maureira D. Orellana-Serradell O. Silva I. Lavanderos B. Cruz P. (2021). TRP channels interactome as a novel therapeutic target in breast cancer. Front. Oncol. 11, 621614. 10.3389/fonc.2021.621614 34178620 PMC8222984

[B77] Sánchez-Hernández R. Benítez-Angeles M. Hernández-Vega A. M. Rosenbaum T. (2024). Recent advances on the structure and the function relationships of the TRPV4 ion channel. Channels (Austin) 18 (1), 2313323. 10.1080/19336950.2024.2313323 38354101 PMC10868539

[B78] Santoni G. Amantini C. Maggi F. Marinelli O. Santoni M. Nabissi M. (2020). The TRPV2 cation channels: from urothelial cancer invasiveness to glioblastoma multiforme interactome signature. Lab. Invest 100 (2), 186–198. 10.1038/s41374-019-0333-7 31653969

[B79] Santoni G. Farfariello V. Amantini C. (2011). TRPV channels in tumor growth and progression. Adv. Exp. Med. Biol. 704, 947–967. 10.1007/978-94-007-0265-3_49 21290335

[B80] Santoni G. Maggi F. Morelli M. B. Santoni M. Marinelli O. (2019). Transient receptor potential cation channels in cancer therapy. Med. Sci. (Basel). 7 (12), 108. 10.3390/medsci7120108 31801263 PMC6950741

[B81] Shu S. Xu Y. Zhan Q. (2021). Understanding metabolic reprogramming in tumor microenvironment. Med. Rev. 1, 111–113. 10.1515/mr-2021-0037 PMC10388741 37724298

[B82] Song C. Bae Y. Jun J. Lee H. Kim N. D. Lee K. B. (2017). Identification of TG100-115 as a new and potent TRPM7 kinase inhibitor, which suppresses breast cancer cell migration and invasion. Biochim. Biophys. Acta Gen. Subj. 1861 (4), 947–957. 10.1016/j.bbagen.2017.01.034 28161478

[B83] Stokłosa P. Borgström A. Kappel S. Peinelt C. (2020). TRP channels in digestive tract cancers. Int. J. Mol. Sci. 21 (5), 1877. 10.3390/ijms21051877 32182937 PMC7084354

[B84] TaiYK C. K. K. W. Fong C. H. H. Ramanan S. Yap J. L. Y. Yin J. N. Yip Y. S. (2022). Modulated TRPC1 expression predicts sensitivity of breast cancer to doxorubicin and magnetic field therapy: segue towards a precision medicine approach. Front. Oncol. 11, 783803. 10.3389/fonc.2021.783803 35141145 PMC8818958

[B85] Thompson E. G. Schaheen L. Dang H. Fares H. (2007). Lysosomal trafficking functions of mucolipin-1 in murine macrophages. BMC Cell Biol. 8, 54. 10.1186/1471-2121-8-54 18154673 PMC2254603

[B86] Vercelli C. Barbero R. Cuniberti B. Odore R. Re G. (2015). Expression and functionality of TRPV1 receptor in human MCF-7 and canine CF.41 cells. Vet. Comp. Oncol. 13 (2), 133–142. 10.1111/vco.12028 23510405

[B87] Wang C. Wang J. Zhao L. Wang Y. Liu J. Shi L. (2008). Sildenafil inhibits human pulmonary artery smooth muscle cell proliferation by decreasing capacitative Ca^2+^ entry. J. Pharmacol. Sci. 108 (1), 71–78. 10.1254/jphs.08069fp 18818482

[B88] Wang Q. Guo W. Hao B. Shi X. Lu Y. Wong C. W. (2016). Mechanistic study of TRPM2-Ca^2+^-CAMK2-BECN1 signaling in oxidative stress-induced autophagy inhibition. Autophagy 12 (8), 1340–1354. 10.1080/15548627.2016.1187365 27245989 PMC4968236

[B89] Wang X. Li G. Zhang Y. Li L. Qiu L. Qian Z. (2022a). Pan-cancer analysis reveals genomic and clinical characteristics of TRPV channel-related genes. Front. Oncol. 12, 813100. 10.3389/fonc.2022.813100 35174089 PMC8841404

[B90] Wang Y. Lu R. Chen P. Cui R. Ji M. Zhang X. (2022b). Promoter methylation of transient receptor potential melastatin-related 7 (TRPM7) predicts a better prognosis in patients with LuminalA breast cancers. BMC Cancer 22 (1), 951. 10.1186/s12885-022-10038-z 36064388 PMC9446581

[B91] Wang Y. Qi Y. X. Qi Z. Tsang S. Y. (2019). TRPC3 regulates the proliferation and apoptosis resistance of triple negative breast cancer cells through the TRPC3/RASA4/MAPK pathway. Cancers (Basel) 11 (4), 558. 10.3390/cancers11040558 31003514 PMC6520729

[B92] Weber L. V. Al-Refae K. Wölk G. Bonatz G. Altmüller J. Becker C. (2016). Expression and functionality of TRPV1 in breast cancer cells. Breast Cancer (Dove Med. Press) 8, 243–252. 10.2147/BCTT.S121610 28008282 PMC5167528

[B93] Wong K. K. Hussain F. A. (2020). TRPM4 is overexpressed in breast cancer associated with estrogen response and epithelial-mesenchymal transition gene sets. PLoS One 15 (6), e0233884. 10.1371/journal.pone.0233884 32484822 PMC7266295

[B94] Wu T. T. Peters A. A. Tan P. T. Roberts-Thomson S. J. Monteith G. R. (2014). Consequences of activating the calcium-permeable ion channel TRPV1 in breast cancer cells with regulated TRPV1 expression. Cell Calcium 56 (2), 59–67. 10.1016/j.ceca.2014.04.006 24889371

[B95] Xu X. Li N. Wang Y. Yu J. Mi J. (2021). Calcium channel TRPV6 promotes breast cancer metastasis by NFATC2IP. Cancer Lett. 519, 150–160. 10.1016/j.canlet.2021.07.017 34265397

[B96] Xu Y. Wu X. Li F. Huang D. Zhu W. (2018). CDCA4, a downstream gene of the Nrf2 signaling pathway, regulates cell proliferation and apoptosis in the MCF - 7/ADM human breast cancer cell line. Mol. Med. Rep. 17 (1), 1507–1512. 10.3892/mmr.2017.8095 29257222 PMC5780089

[B97] Yapa KTDS Deuis J. Peters A. A. Kenny P. A. Roberts-Thomson S. J. Vetter I. (2018). Assessment of the TRPM8 inhibitor AMTB in breast cancer cells and its identification as an inhibitor of voltage gated sodium channels. Life Sci. 198, 128–135. 10.1016/j.lfs.2018.02.030 29496495

[B98] Yuan J. P. Zeng W. Dorwart M. R. Choi Y. J. Worley P. F. Muallem S. (2009). SOAR and the polybasic STIM1 domains gate and regulate Orai channels. Nat. Cell Biol. 11 (3), 337–343. 10.1038/ncb1842 19182790 PMC2663385

[B99] Zhang H. Zhang X. Wang X. Sun H. Hou C. Yu Y. (2022). Comprehensive analysis of TRP channel-related genes in patients with triple-negative breast cancer for guiding prognostic prediction. Front. Oncol. 12, 941283. 10.3389/fonc.2022.941283 35875096 PMC9300844

[B100] Zhang L. Y. Zhang Y. Q. Zeng Y. Z. Zhu J. L. Chen H. Wei X. L. (2020). TRPC1 inhibits the proliferation and migration of estrogen receptor-positive Breast cancer and gives a better prognosis by inhibiting the PI3K/AKT pathway. Breast Cancer Res. Treat. 182 (1), 21–33. 10.1007/s10549-020-05673-8 32415497

[B101] Zhang W. Tang X. Peng Y. Xu Y. Liu L. Liu S. (2024). GBP2 enhances paclitaxel sensitivity in triple-negative breast cancer by promoting autophagy in combination with ATG2 and inhibiting the PI3K/AKT/mTOR pathway. Int. J. Oncol. 64 (4), 34. 10.3892/ijo.2024.5622 38334171 PMC10901536

[B102] Zheng A. Kallio A. Härkönen P. (2007). Tamoxifen-induced rapid death of MCF-7 breast cancer cells is mediated via extracellularly signal-regulated kinase signaling and can be abrogated by estrogen. Endocrinology 148 (6), 2764–2777. 10.1210/en.2006-1269 17363451

[B103] Zheng L. Chen J. Ma Z. Liu W. Yang F. Yang Z. (2016). Capsaicin enhances anti-proliferation efficacy of pirarubicin via activating TRPV1 and inhibiting PCNA nuclear translocation in 5637 cells. Mol. Med. Rep. 13 (1), 881–887. 10.3892/mmr.2015.4623 26648574

